# Identification of nonlinear features in cortical and subcortical signals of Parkinson's Disease patients via a novel efficient measure

**DOI:** 10.1016/j.neuroimage.2020.117356

**Published:** 2020-12

**Authors:** Tolga Esat Özkurt, Harith Akram, Ludvic Zrinzo, Patricia Limousin, Tom Foltynie, Ashwini Oswal, Vladimir Litvak

**Affiliations:** a Wellcome Trust Centre for Neuroimaging, UCL Institute of Neurology, London, UK; b Middle East Technical University, Department of Health Informatics, Graduate School of Informatics, Ankara, Turkey; c Department of Clinical and Movement Neurosciences, UCL Institute of Neurology and The National Hospital for Neurology and Neurosurgery, Queen Square, London, UK; d Department of Clinical Neurology, John Radcliffe Hospital, Oxford, UK

**Keywords:** Deep brain stimulation, Dopamine, Levodopa, Local field potentials, Neural oscillations, Nonlinearity

## Abstract

This study offers a novel and efficient measure based on a higher order version of autocorrelative signal memory that can identify nonlinearities in a single time series. The suggested method was applied to simultaneously recorded subthalamic nucleus (STN) local field potentials (LFP) and magnetoencephalography (MEG) from fourteen Parkinson's Disease (PD) patients who underwent surgery for deep brain stimulation. Recordings were obtained during rest for both OFF and ON dopaminergic medication states. We analyzed the bilateral LFP channels that had the maximum beta power in the OFF state and the cortical sources that had the maximum coherence with the selected LFP channels in the alpha band. Our findings revealed the inherent nonlinearity in the PD data as subcortical high beta

(20–30 Hz) band and cortical alpha (8–12 Hz) band activities. While the former was discernible without medication (*p*=0.015), the latter was induced upon the dopaminergic medication (*p*<6.10^−4^). The degree of subthalamic nonlinearity was correlated with contralateral tremor severity (*r*=0.45, *p*=0.02). Conversely, for the cortical signals nonlinearity was present for the ON medication state with a peak in the alpha band and correlated with contralateral akinesia and rigidity (*r*=0.46, *p*=0.02). This correlation appeared to be independent from that of alpha power and the two measures combined explained 34 % of the variance in contralateral akinesia scores. Our findings suggest that particular frequency bands and brain regions display nonlinear features closely associated with distinct motor symptoms and functions.

## 1 Introduction

Abnormal oscillatory brain activity is considered to be a prominent feature of Parkinson's disease (PD). It can be detected in subcortical local field potentials (LFP) collected from electrodes implanted during deep brain stimulation (DBS) surgery as well as in cortical signals acquired as EEG and MEG (magnetoencephalography) measurements (for a recent review see Geraedts et al., 2018 for EEG and Boon et al., 2019 for MEG). It is possible to collect LFP and MEG signals simultaneously in patients with temporarily externalized electrode leads. MEG is uniquely suitable for such recordings because the sensors do not come in direct contact with the skin, which is critical for patients immediately after surgery. Furthermore, MEG measurements allow for cortical source identification with high spatial resolution as magnetic fields are not distorted by head tissues.

The subthalamic nucleus (STN) is the most common DBS target in PD. Simultaneous MEG and STN-LFP recordings (Litvak et al., 2010 and Hirschmann et al., 2011) demonstrated that the linear interactions between cortex and STN are realized via two main networks belonging distinctly to alpha and beta frequency bands. The former network was predominantly constrained to the temporal-parietal regions in cortex, while the latter was specific to the mesial sensorimotor regions.

For subcortical regions, abnormal synchronization associated with PD has been most clearly shown in the beta band 13 – 30 Hz (Priori et al., 2004; Brown, 2006; Kühn et al., 2006). It has been demonstrated numerous times that this abnormal beta activity is suppressed by dopaminergic medication (Brown et al., 2001; Priori et al., 2004; Özkurt et al., 2011; West et al. 2016), voluntary movement (Cassidy et al., 2002; Hirschmann et al., 2013a) or DBS applied over 100 Hz (Eusebio et al. 2011; Abbasi et al., 2018). A functional subdivision of beta band into low (13–22 Hz) and high (22–30 Hz) frequency bands has been supported by several LFP studies in PD (e.g., Priori et al., 2004; Little et al., 2012; van Wijk et al., 2016, West et al. 2016).

Apart from the prominent beta band activity described as antikinetic in PD, various other spectral features were investigated such as prokinetic gamma activity of about 70–90 Hz (Brown et al, 2001; Fogelson et al., 2005), high gamma band activity over 100 Hz particularly in the motor cortex (de Hemptinne et al., 2013; Cole et al., 2017) and very high frequency oscillations in the range of 200–400 Hz (Foffani et al., 2003; López-Azcárate et al., 2010; Özkurt et al., 2011). These spectral activities of neural populations have been shown to have interrelations via phase – phase (Schnitzler and Gross, 2005; Hirschmann et al., 2013a) and phase – amplitude couplings (López-Azcárate et al., 2010; Özkurt et al., 2011; de Hemptinne et al., 2015) between MEG cortical sources and STN LFP's (Litvak et al., 2010; Hirschmann et al., 2011) and between different parts of the STN (Hohlefeld et al., 2013; West et al., 2016).

The spectral power in various bands and oscillatory coupling measures such as coherence are derived from Fourier like methods that decompose the signals into sinusoids. These measures depend upon *linear* characteristics of the underlying signal. A vast majority of brain signal analyses in the aforementioned literature have employed methods limited to second-order statistics. However, the brain is well-accepted to be a nonlinear and complex system naturally producing nonlinear and constantly changing nonstationary signals (Bermudez and Laencina, 2015).

Nonlinear properties may be extracted by relying on theoretical frameworks of higher order statistics (HOS) along with higher order polyspectra (Mendel, 1991) and within the context of dynamical system theory (Rijlaarsdam et al., 2017). Extraction of nonlinear features inherent in the brain signals has increasingly gained impetus as a way to characterize the dynamics of basal ganglia circuitry and interpret the neuronal dysfunctions underlying Parkinsonian motor symptoms (Darbin et al., 2013). The presence of nonlinearity in the basal ganglia of PD patients was demonstrated in micro neuronal level through an entropy-based measure applied on the inter-spike intervals (Lim et al., 2010). Müller et al. (2001) utilized the measure of correlation dimension derived from the nonlinear dynamics and showed EEG channel-level topographical differences between PD patients and healthy controls for resting state and motor tasks. An EEG study by Lainscsek et al. (2013a) used a model based on delay differential equations in order to distinguish medication OFF state from the ON state of PD patients. Their derived features correlated with motor impairment.

An indirect but intuitive way to identify nonlinearities is the investigation of oscillatory temporal waveform shapes that deviate from Fourier-like base functions, i.e., sinusoids (Cole and Voytek, 2018). Cole et al. (2017) demonstrated the asymmetry of sharpness of cortical beta band oscillations in PD and how this sharpness is smoothed out upon the application of DBS. Another study by Jackson et al. (2019) applied the waveform shape analysis on scalp EEG signals of PD patients and showed that beta band cycles over sensory motor regions had greater sharpness and steepness asymmetries for the dopaminergic medication OFF state when compared to ON state.

Interestingly, these asymmetric wave shape properties are intimately related to the bispectral signal features (Elgar, 1987; Bartz et al., 2019). Applications of HOS measures, especially 3rd order bispectra and bicoherence, in medicine and biology including brain signal analysis have been widespread (Pradhan et al., 2012; Özkurt, 2016). However, despite several attempts (Marceglia et al., 2006; Özkurt et al., 2017), measures of bispectrum and bicoherence could not be shown to be systematically related to the clinical state in PD. Even though, HOS of EEG signals can provide robust bispectral features for conditions such as the depth of anesthesia and are used practically and efficiently in clinics (Hajat et al., 2017), extending their use to other applications has not proven promising.

In this study, we introduce and develop a novel computationally efficient nonlinear measure based on a higher order version of autocorrelative signal memory. We initially hypothesize that PD STN time series contain nonlinear components that reflect abnormal information load and the dopaminergic medication diminishes this effect. To test this hypothesis, we apply our measure in order to identify nonlinear properties both in STN LFP and in signals from the cortical sources coherent with the STN at rest. Specifically, we test whether the suggested measure is affected by dopaminergic medication and whether it is correlated with the clinical impairment.

## 2 Methods

### 2.1 nsAMDF: a novel nonlinear metric of nonlinearity

In this section, we formulate and present our novel metric that can identify nonlinearity from a single time-series.

Average magnitude difference function (AMDF) was initially defined and utilized to estimate the pitch period of speech signals (Ross et al., 1974). It was suggested as an alternative to the autocorrelation function:

(1) $$D_{\tau }=\frac{1}{L}\sum_{j=1}^{L}\left|x_{j}-x_{j-\tau }\right|,\;\;\;\;\;\;\;\;\tau =0,1,...,\tau_{\max}$$

where *L* is data length, τ is the lag, as *x* and *D* denote the signal and AMDF, respectively. It was shown to be approximately related to the autocorrelation *R* simply as:

(2) $$D_{\tau }≅a{\left[2(R_{0}-R_{n})\right]}^{1/2}.$$

Here *a* is a constant value varying from 0.6 to 1 and *R_0_* is the autocorrelation at 0. Notice that AMDF and autocorrelation are reciprocal, i.e., they are very strongly negatively correlated. Both of these functions indicate linear 2nd order memory of the signal. A short-time AMDF was introduced by Akgül et al. (2000) in order to capture sleep spindle related regularities from sleep EEG signals

(3) $$D_{\tau }\left(n\right)=\frac{1}{M}\sum_{m=1}^{M}\left|x_{n+m}w_{m}-x_{n+m-\tau }w_{m-\tau }\right|$$

where τ is the lag*, M* is the window length and *w_m_* is the window function. This study used AMDF with windowed segments. This is reasonable as EEG data are typically nonstationary.

Here we introduce a slight but important modification to this measure, by adding an integer-wise “degree” *p* and defining it in terms of norms. This change in AMDF radically transforms the measure to identify nonlinear properties and components of the underlying signal:

(4) $$s_{x}\left(\tau ,p\right){=}^{1/p}\sqrt{E\left\{{\left|x_{j}-x_{j-\tau }\right|}^{p}\right\}}=E\left\{norm_{p}\left(x_{j},x_{j-\tau }\right)\right\}$$

We will call it nonlinear short-time AMDF (nsAMDF) throughout this text. Interestingly, nsAMDF comprises a linear combination of many special higher-order cumulants or higher order autocorrelations *C*, which are the temporal equivalents of polyspectra. This can be easily seen as follows:

(5) $$\begin{array}{ccc} & & E\left\{{\left[x_{j}-x_{j-\tau }\right]}^{p}\right\}=\sum_{k=0}^{p}{\left(-1\right)}^{p}E\left\{{x_{j}}^{k}{x_{j-\tau }}^{p-k}\right\}\\ & & =\sum_{k=0}^{p}{\left(-1\right)}^{p}C\left(0,0,...,0,\tau ,\tau ,...,\tau \right). \end{array}$$

We suggest that nonlinear and nonstationary properties of PD brain signals can be captured by a very simple and efficiently computable measure defined based on the difference between nsAMDF measures for the degrees of *p*=2 and *p*=7. Please note that the latter degree can be optimized with respect to the properties of a particular signal.

### 2.2 Implementation of nsAMDF and the associated nonlinearity measure

We computed nsAMDF given in Eq. (4) for the norm degrees *p*=2 and *p*=7. The former indicates and is closely related to autocorrelation sequence, hence embodies “linear” characteristics of the signal. The latter, on the other hand, carries information regarding higher order statistics, hence nonlinear properties. While estimating these measures, we took half overlapped segments of approximately 14 s and computed nsAMDF for the maximum lag of 1s. Normalization of nsAMDF is basically realized by dividing all the values by the maximum. Proper estimation of higher-order nonlinear measures such as cumulants and polyspectra requires longer data segments when compared to the second-order measures of autocorrelation and power spectrum. One should also not make them too long to avoid potential nonstationarities inherent in the data (Nikias and Mendel, 1993). Length of data segments should be chosen with a consideration of this trade-off.

We compared these processed and normalized nsAMDF sequences for the degrees of 2 and 7 to identify nonlinearity. Any significant deviation of the latter from the former is accepted as evidence for existence of “nonlinear” component in the signal. We define this deviation as the nonlinearity measure *L* and estimate it as follows:

(6) $$L=\mathrm{norm}\left(s_{x}\left(l,7\right){\;}_{\mathrm{norm}\mathrm{aliz}\mathrm{ed}}-s_{x}{\left(l,2\right)}_{\mathrm{norm}\mathrm{aliz}\mathrm{ed}}\right)$$

Ideally, any nonzero *L* indicates the presence of possible nonlinearity as with an ideal Gaussian signal, *L* would amount to zero considering Eqs. (5) and (6). However, in practice, because of finitude and nonstationarity of the real signals, a confidence level may be required to determine the existence of nonlinearity from *L*, especially in case there is no other condition to compare with. Confidence levels can be empirically determined either by simulation of Gaussian signals with the length of the data underhand or by producing surrogate signals as described in Section 2.3.

In case significant deviation was identified, we estimated the spectra of the normalized sequences of nsAMDF for the degrees of 2 and 7, in order to observe the frequency domain correspondence of the nonlinear memory. Even though nsAMDF has essentially temporal character, one can bandpass-filter the nsAMDF sequences prior to the computation of *L*, if the nonlinearity is identified over an oscillatory band.

Eq. (5) implies that the nsAMDF value accounts for signal components with orders up to the degree *p*. Accordingly, the chosen degree *p* should ideally be large enough to capture the highest order of nonlinearity within the data. However, in practice, one does not know *a priori* what this degree is for the data underhand. We chose *p*=7 for our dataset from observations of *L* for various degrees. In the supplementary figure, one can see how *L* increases and reaches a plateau for a representative patient showing prominent nonlinearity. The relatively weak increment starting with *p*=8 was considered to be reflecting estimation noise. Hence the nsAMDF degree *p*=7 by definition containing all the lower moments up to 7 was adequate to capture nonlinear components inherent to our data. A fixed nonlinearity degree *p* for all subjects is necessary for consistent and fair comparison of group level estimates. One may choose another suitable degree optimized empirically for other types of applications and signals.

Please note that for data length of 14 s, it took about 5.5 s to compute the nonlinearity measure with a basic laptop computer. When the data length increases, the computation time expectedly increases linearly.

### 2.3 Surrogate time series

It has been common to test for inherent nonlinearity using surrogate data preserving all linear characteristics of the data underhand (Schreiber and Schmitz, 2000). The null hypothesis for this test is that the data are generated by a linear Gaussian stochastic process. A number of realizations of the surrogate are produced and the nonlinear measure is applied to these data. If the accepted nonlinear method or measure (in our case nsAMDF) has significant deviations for original data when compared to surrogate data, then the null hypothesis is rejected. In other words, the original data are accepted to have a significant nonlinear structure. If the measure for the original data has a similar probability distribution with the ones obtained from the surrogate data, then the null hypothesis cannot be rejected, i.e., there is no identifiable nonlinear structure in the data.

Accordingly, we used the amplitude adjusted Fourier transform (AAFT) originally proposed by Theiler et al. (1992) in order to produce the surrogates. The surrogates are basically produced from the same Fourier coefficients of the original data. Only the phase is randomized and the time series is forced to conform to a Gaussian probability distribution. This eliminates higher order statistical and nonlinear properties.

## 3 Data

### 3.1 Patients and empirical data

**Fig. 1:**
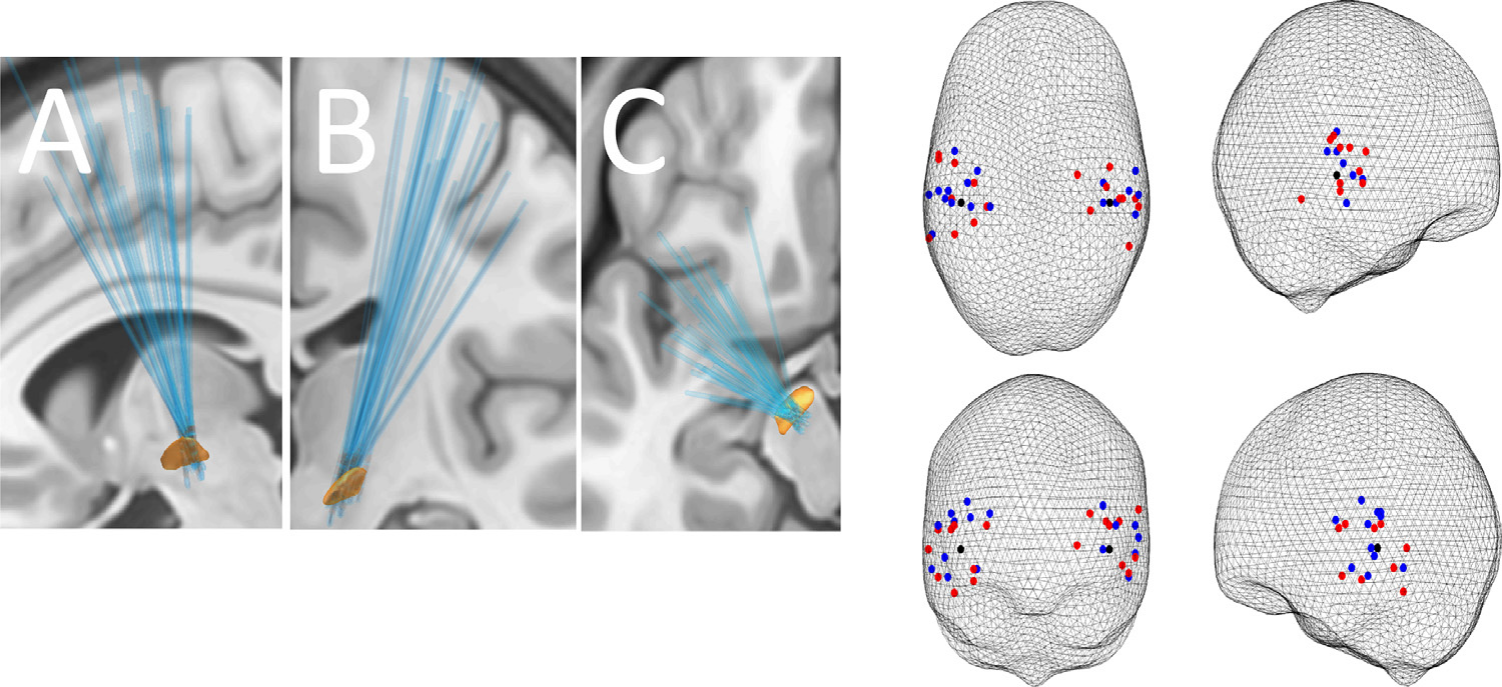
**(Left)** Reconstructed electrode locations in sagittal- view from the left (A), coronal-view from the front (B) and axial- view from the top (C) projections. All the 28 electrodes from both hemispheres are shown together in relation to left STN outline based on the DISTAL atlas (Ewert et al., 2018). **(Right)** Locations of cortical sources coherent with the STN in the alpha band which were used for the analysis of cortical activity. The sources were identified as the local peaks in DICS coherence images closest to the group peak previously reported by Litvak et al. (2011) MNI coordinates [46, −30, −2] on the right and the symmetric location on the left shown as black circles. The DICS images corresponded to those STN channels that were used for the LFP analysis. Peaks were identified separately for ON drug (blue) and OFF drug (red) condition. In cases where there was no local peak withing 30 mm radius from the group peak, the extraction was done from the group peak (19/56 extractions).

STN LFP and MEG data were taken from the study by Litvak et al. (2012) with two additional subjects previously also reported by West et al. (2016). They were acquired from 14 PD patients (3 female) who underwent DBS surgery, while their implanted DBS electrodes were externalized postoperatively. The patient details are provided in Table 1 and reconstructed electrode locations are shown in Fig. 1 (left panel).

**Table 1:** **Details of DBS patient cohort.** All patients have PD and have been treated with deep brain stimulation (DBS) targeted to the STN. The patients’ age, gender, duration of PD since diagnosis, predominant clinical features, unified Parkinson's disease rating scale (UPDRS, 2008 part III-Motor Impairment) ON and OFF medication and LFP channels selected for analysis are shown. The UPDRS score is out of a maximum of 104 with higher scores indicative of increased severity.

ID	Age (years)	Sex	Disease Duration (years)	Predominant Symptoms	Motor UPDRS (ON/OFF)	Pre-operative Medication	STN channel analysed Left/Right
1	58	M	13	Gait Freezing	25/43	Co-careldopa (100/25 × 8) 1000mg Co-careldopa modified release (100/25) CR 125mg Amantadine 400mg Co-beneldopa (100/25) 125mg Entacapone 600mg Rasagiline 1mg Ropinirole 6mg	23/01
2	57	M	17	Gait Impariment, pain, dyskinesias	14/54	Co-careldopa (100/25 × 9) 1125mg Co-careldopa modified release (200/50) 250mg Co-beneldopa (200/50) 250mg Entacapone 1600mg Selegiline 10mg Amantadine 200mg	01/12
3	60	M	15	Dyskinesia, gait freezing	10/56	Co-careldopa (100/25 × 9) 1125mg Co-beneldopa (200/50) 250mg Ropinirole 18mg Selegiline 10mg Amantadine 200mg	12/23
4	48	M	11	Gait freezing, tremor	16/72	Rasagiline 1mg Co-careldopa (100/25 × 10) 1250mg Entacapone 1000mg Pramiprexole 0.18 mg	12/12
5	52	M	12	Dystonia	10/35	Rotigotine 4mg Stalevo (100/25/200 × 7 + 50/12.5/200 × 5) 950mg Rasagiline 1mg	12/23
6	58	F	10	Dystonia, dyskinesia	16/55	Pramipexole 2.1mg Stalevo (100/25/200 × 4 + 50/12.5/200) 450mg Rasagiline 1mg Co-careldopa (100/25)	23/12
7	55	M	15	Tremor, freezing	5/19	Co-beneldopa (200/50 × 4) 1000mg Ropinirole16mg Selegiline10mg Amantadine100mg	12/12
8	58	F	14	Gait freezing, pain, dyskinesias	18/71	Pramipexole 2.8mg Stalevo (200/50/200)mg Amantidine 100mg	23/23
9	62	M	9	Off periods + dyskinesia	28/5	Co-careldopa (100/25 × 14) 1750 mg Amantidine 300mg Entacapone 800mg Pramipexole 0.35 mg	01/01
10	51	M	8	Off periods + FOG	49/21	Co-beneldopa (100/25 × 3 + 200/50 + 50/12.5 × 2) 750 mg Rasagiline 1mg	23/01
11	61	F	7	Gait disturbance	35/4	Stalevo (150/37.5/200)x2 - 300mg Co-careldopa (100/25) + (50/12.5)x3 + (200/50) 450 mg	23/23
12	54	M	15	Off periods	53/19	Co-careldopa (100/25 × 10) 1250mg Pramipexole 1.05mg	23/12
13	40	M	6	Off periods + dyskinesias	30/9	Stalevo (150/37.5/200) x 4 - 600mg Co-careldopa (100/25 × 3 + 200/50) – 625mg Pramipexole 3.15 mg	23/12
14	54	M	8	Off periods + dyskinesias	38/9	Co-careldopa (100/25) x 12 Cabergoline 6mg entacapone 1000mg Amantidine 400mg	23/23

The patients’ motor symptoms were evaluated based on the standard Unified Parkinson's Disease Rating Scale (UPDRS III) prior to the surgery. The data acquisition procedure was approved by the joint ethics committee of the National Hospital for Neurology and Neurosurgery and the University College London (UCL) Institute of Neurology, and the patients provided written informed consent.

Bilateral STN LFP and MEG eyes open resting state recordings were done twice in each patient (usually on different days). One recording was done after overnight withdrawal of their dopaminergic medication (OFF state) and the other after taking their usual medication dose (ON state). The order of the two recordings was counterbalanced across patients.

MEG recordings were obtained with a 275-channel system (CTF/VSM MedTech, Vancouver, Canada). The sampling frequency was 2400 Hz and the analyzed data length was approximately 3 min for each condition. Bipolar LFP channels (01, 12, 23) were constructed by referencing the adjacent contacts to each other. Our analysis only used the LFP channels that had the highest beta power in the OFF state for each nucleus. Numerical routines were written and data analyses, statistics and plotting were performed using the numerical software platform of Matlab® version 2018 (Mathworks Inc., Natick, MA, USA).

### 3.2 Preprocessing

The data were pre-processed using open source SPM (Litvak et al., 2011) and Fieldtrip (Oostenveld et al., 2011) toolboxes. The continuous resting recording was converted to SPM format. Following conversion, the LFP recording originally acquired with the right mastoid as the reference were converted to bipolar montage between adjacent contacts (3 channels per hemisphere). Flat segments exceeding 10 consecutive samples and discontinuous jumps exceeding 20 pT were marked in the MEG channels prior to any processing and the segments containing these artefacts were later excluded. Digital filtering was then applied to remove frequency component below 1 Hz and 50 Hz line noise and its harmonics up to 600 Hz (5th order, two-pass Butterworth filters). The filtered data were epoched into 3.4 s segments and segments containing previously marked artefacts in the MEG or deflections in the LFP exceeding a threshold, adjusted separately for each subject based on visual inspection of the LFP data, were rejected. Prior to nsAMDF calculation, the data were additionally low-pass filtered with the cut-off frequency of 40 Hz by two-way least-squares finite impulse response filtered signals using *eegfilt* routine from EEGLAB toolbox (Delorme and Makeig, 2004).

### 3.3 Cortical source activity estimation from MEG data

Cortical sources maximally coherent with STN LFP alpha band activities were located using the dynamic imaging of coherent sources (DICS) beamforming method (Gross et al., 2001). Subsequently, time series were extracted from individual peak locations closest to the group peaks reported in Litvak et al (2011). In cases where there was no individual peak within 30 mm radius from the group peak (19/56 extractions), the group peak was chosen instead (Fig. 1, right panel). Virtual electrode time series for these source locations were obtained using time-domain linearly constrained minimum variance (LCMV) beamformer (VanVeen et al., 1997). We refer the reader to Litvak et al. (2010) for the details of cortical source reconstruction methodology.

### 3.4 Spectral analysis

The spectral power of STN-LFP and cortical signals was estimated using multitapering method (Thomson, 1982) with frequency resolution of ±1 Hz. The spectra were averaged across epochs and normalised using “Fitting Oscillations and One Over F” (FOOOF; Haller et al., 2018) algorithm to emphasize and isolate their oscillatory components. FOOOF decomposes a spectrum into a non-oscillatory ‘background’ component modelled as an exponential with a ‘knee’ and a set of oscillatory components modelled as Gaussians. The STN-LFP spectra were fitted in the 1–95 Hz range with the filter notch in 45–55 Hz range replaced by linear interpolation. The individual fits were visually inspected to ensure their quality. Since the numbers and central frequencies of the Gaussians varied between different, we only used FOOOF to subtract the background component. The residuals of this subtraction were then treated as normalized log-spectra.

### 3.5 Simulated data

**Fig. 2:**
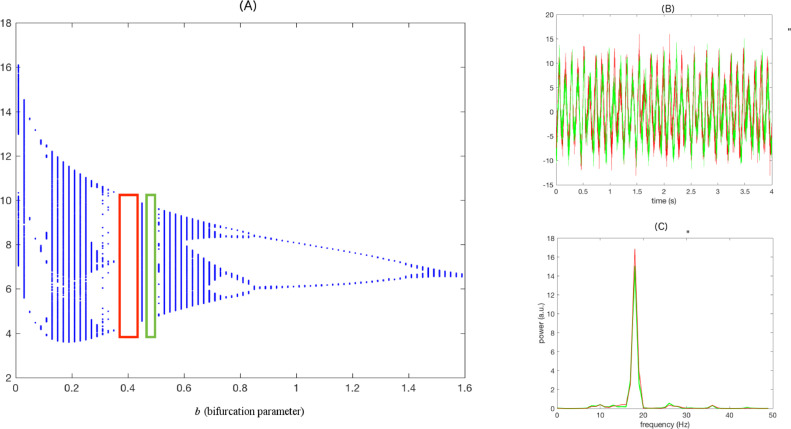
(A) Rössler system bifurcation diagram and the chosen bifurcation parameters for the simulation. Example of (B) simulated signals and (C) their corresponding power spectra (Colors indicate the simulated conditions. red: OFF and ON: green).

We simulated time series in order to check the discriminatory power of our suggested metric nsAMDF to identify inherent nonlinearity. To this end, we employed the method presented by Lainscsek et al. (2013b) in order to simulate nonlinear time series that mimic PD-related electrophysiological dynamics. Accordingly, time series *x*(*t*) were generated by the Rössler system

$$\dot{x}=-y-z$$

$$\dot{y}=x+ay$$

$$\dot{z}=b-cz+xz$$

with all parameters fixed except the bifurcation parameter *b*. A unique bifurcation parameter was assigned to each subject. As suggested by Lainscsek et al. (2013b), we selected *b* such that it reflects the wider range of variability indicating PD characteristics. Both PD OFF and PD ON states consisted of 10 subjects with 10 different bifurcation parameters (Fig. 2) as follows: b_OFF = [0.37 0.385 0.39 0.4 0.41 0.42 0.425 0.43 0.435 0.44] b_ON = [0.46 0.4625 0.465 0.47 0.475 0.48 0.4825 0.485 0.4875 0.49].

Note that these selected bifurcation parameters were kept very similar to those used by Lainscsek et al. (2013b) for characterizing PD dynamics. Step size was chosen as 0.05. SNR was taken as 10 dB and the other bifurcation parameters *a* and *c* were kept fixed as 0.2 and 5.7, respectively, to locate the dynamical system in the chaotic range as originally practiced by Rössler (1976). The sampling frequency was 2048 Hz. The first 100,000 data points of *x*(*t*) were discarded to eliminate the effects arising from the initial conditions. Data length was taken as 100,000.

## 4 Results

### 4.1 Simulations

**Fig. 3:**
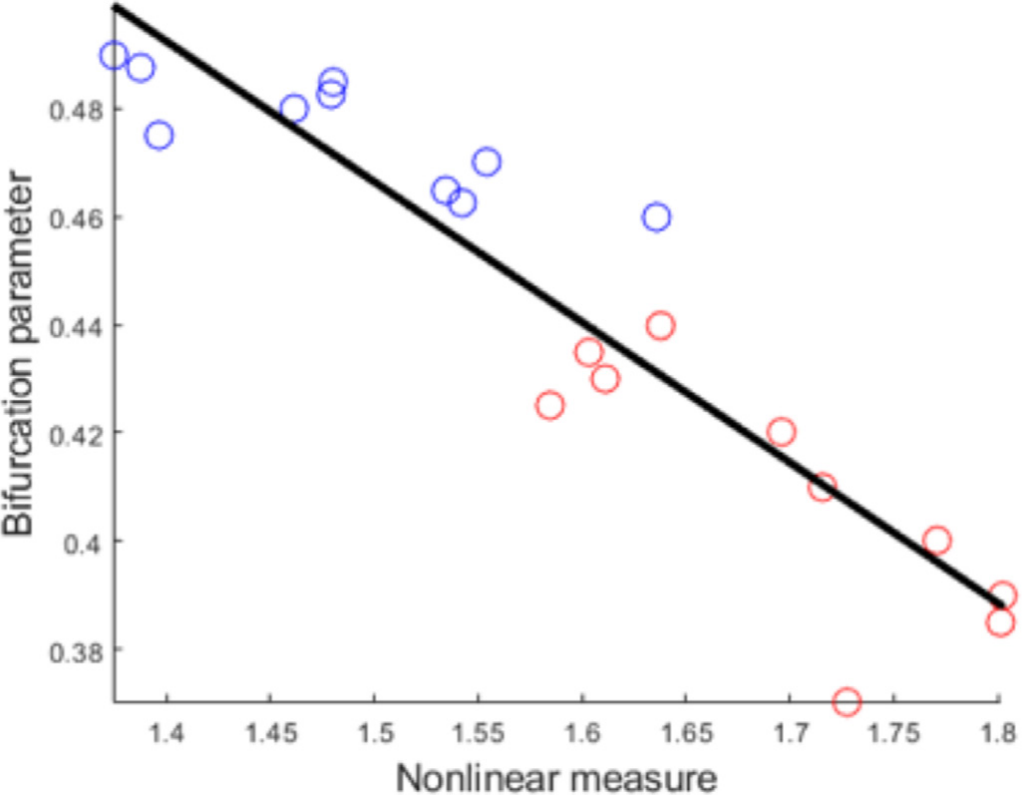
Correlation between bifurcation parameter and nonlinear measure of the simulated signals. The red dots indicate the nonlinear measure obtained for OFF state and the blue dots indicate that for ON state.

nsAMDF based nonlinear measure *L* could well separate the simulated PD OFF and ON states (paired *t* test, *N* = 10, *p* < 10^−14^). There was also tight correlation between the bifurcation parameter *b* indicating the correspondence of complex system characteristics and the nonlinear measure *L* (Fig. 3; *r* = −0.9235, *p* < 10^−8^).

### 4.2 High beta nonlinearity in the STN LFP

We hypothesized that there exists nonlinear memory component inherent in STN-LFP time series in PD and this component is expected to be suppressed by dopaminergic medication. The reasoning was that pathological activity within the PD motor networks may have a nonlinear part increasing the information load. Dopaminergic medication is expected to reduce this possible effect.

STN channels with the highest peak in the beta (13–30Hz) band in the OFF medication state was selected for analysis from each hemisphere. The peaks were identified in log-spectra following subtraction of the non-oscillatory spectral component (see Methods).

**Fig. 4:**
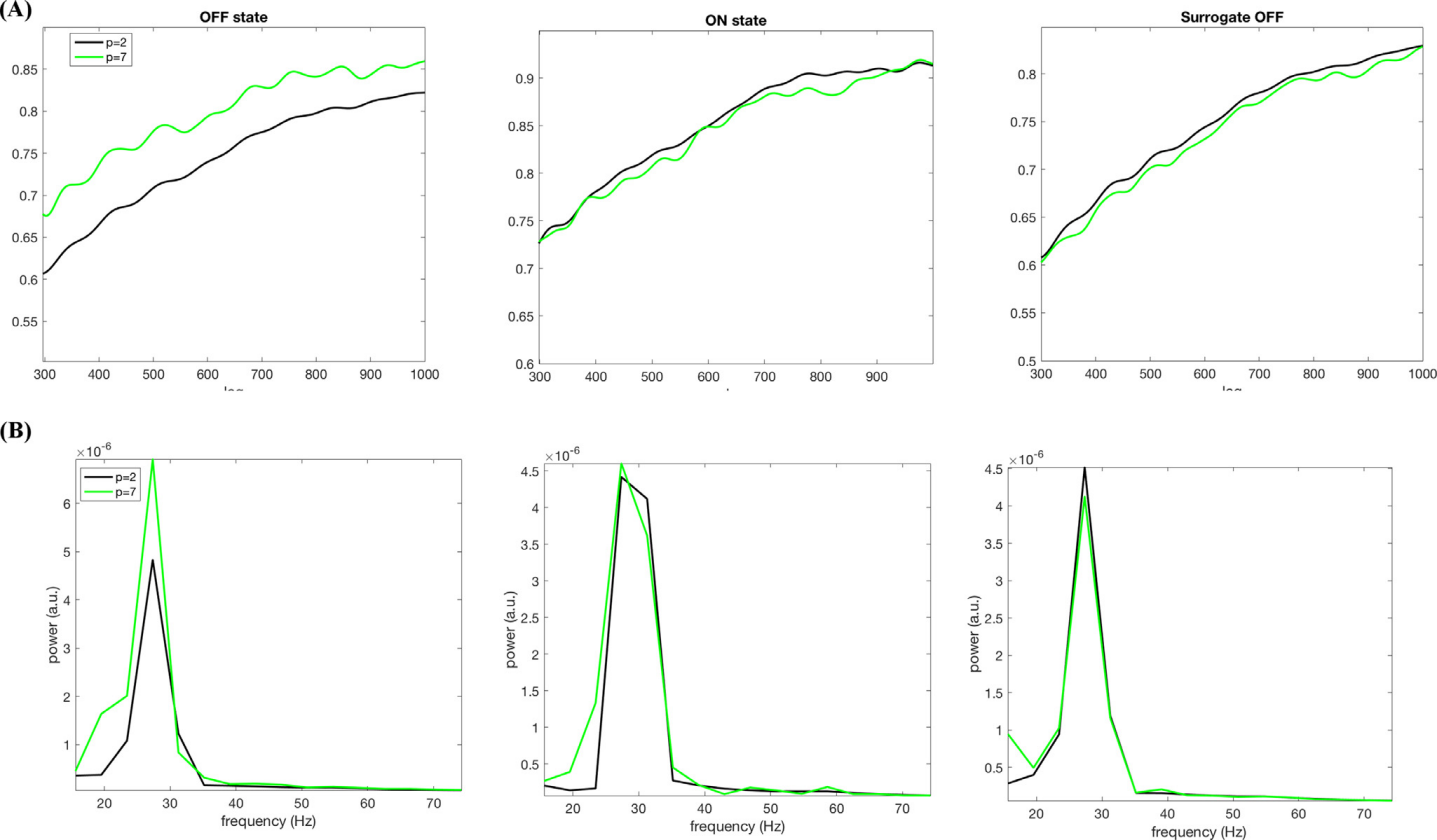
(A) nsAMDF for the degrees *p*=2 (black) and *p*=7 (green) for LFP OFF state, ON state and OFF state surrogate time series for a representative subject and (B) their corresponding spectra in the same order of states.

Fig. 4(A) (left column) demonstrates that the nsAMDF for *p*=7 representing the nonlinear memory contains a discernible ripple, which it is lacking for the linear memory (*p*=2) for a representative subject. Interestingly, upon dopaminergic medication, this effect disappears (middle column). Quantitatively, the nonlinearity measure *L* decreases from 2.29 (OFF state) to 0.58 (ON state) upon medication.

The frequency of the “nonlinear” ripple can easily be identified by observing peak-to-peak of the difference between the normalized nsAMDF of degrees 2 and 7 in Fig. 4(A). Fourier analysis shows that it is focused in a narrow spectral band centered at about 27 Hz (Fig. 4(B)), which belongs to the “high” beta band commonly observed in PD LFP studies (Priori et al., 2004) lying over the range of 20–30 Hz. This sub-band activity is known to have the highest coherence with the motor cortex (Hirschmann et al., 2011; Litvak et al., 2010).

Note that the beta ripple for OFF state disappears after eliminating the higher-order nonlinear terms using AAFT surrogate data production technique, which keeps the second order spectral properties of the data intact (Fig. 4, the right column). Comparing the left and middle columns of Fig. 4(B) for *p*=2 implies that the original and surrogate data have very similar linear memory sequences, showing that the beta ripple stems from nonlinearity belonging to the OFF state LFP signal. The nonlinearity parameter *L* becomes 0.5 for OFF state surrogate time series, nearing to the metric computed for ON state being a way lower than for the OFF state (*L* = 2.29). This also confirms that the observed difference between nsAMDF for *p*=2 and *p*=7 stems from nonlinearity within the LFP signal for the OFF state.

**Fig. 5:**
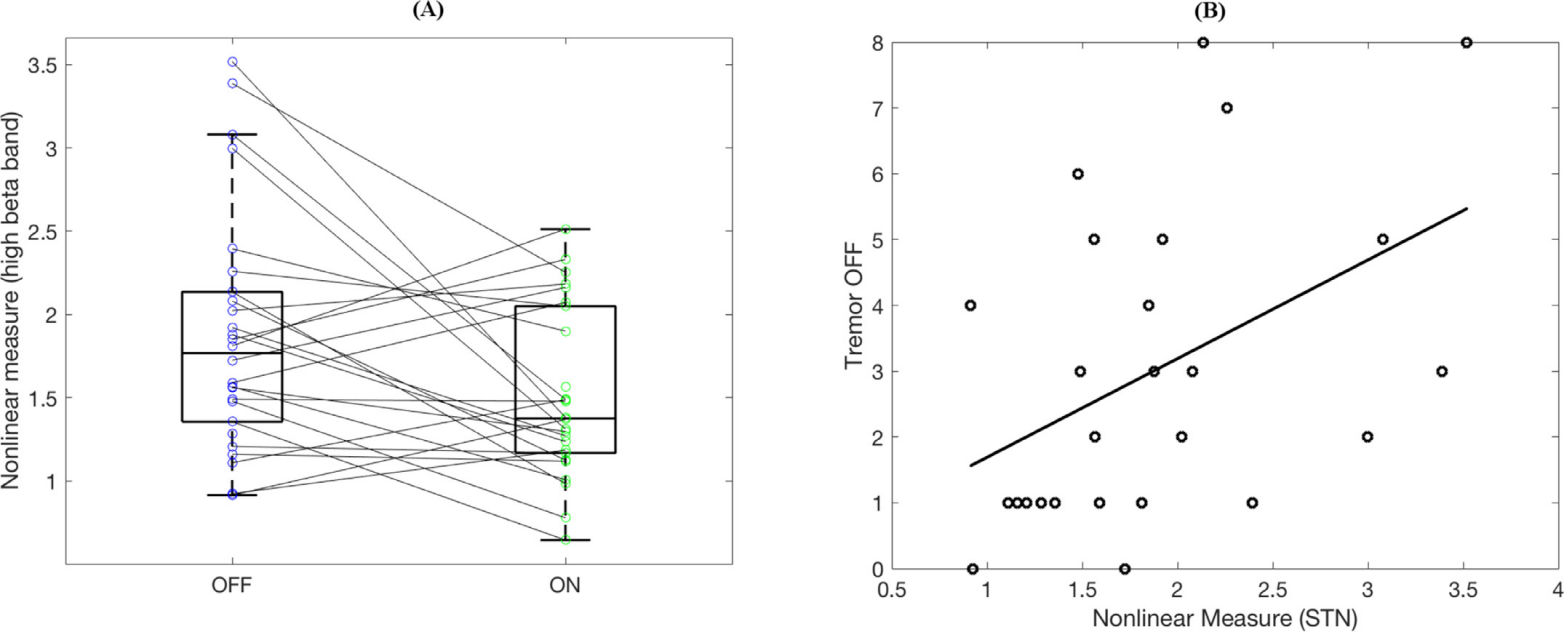
(A) Nonlinearity is higher for OFF than that is for ON state for STN LFP in group level (*N*=26; *p* = 0.015). (B) The nonlinearity measure correlates with the tremor scores in the OFF state (*r*=0.45, *p* = 0.02).

Statistical analysis over the whole group (Fig. 5(A)) showed that the nonlinearity measure *L* was significantly higher for the OFF state than the ON state for the bilateral LFP contacts (paired *t* test, *N*=26; *p* = 0.015). Two nonlinear measure estimates falling out of 95 % of the range were taken as outliers and removed from the analysis. Interestingly, the nonlinearity measure *L* for high beta band correlated with contralateral UPDRS tremor scores (Fig. 5(B); Pearson's correlation, *r*=0.45, *p* = 0.02). It did not have any significant correlation with akinesia scores (*r*=0.23, *p* = 0.36).

Please note that nine sides (out of twenty-six), who showed an opposing pattern of nonlinearity, had also lower tremor scores (1.55) when compared to the rest of the subthalamic nuclei (3.57). Tremor OFF – Tremor ON delta score describing the effect of dopaminergic medication on the former group was also lower (1.22) than that of the rest (3.22). Two subjects who had zero tremor without the medication belonged to the former group. Hence, for this minority subset of patients, the lack or slight existence of tremor is probably the main factor behind the lack of nonlinearity in the OFF state when compared to the ON state.

To compare the clinical correlations of the nonlinear measure with those of beta power we also computed low-beta power estimates by averaging the corrected log-spectra (see Section 2) in the 13–22Hz range for the OFF medication state. When testing the same 26 sides as used for correlations with the nonlinear measure, the beta values were significantly correlated with contralateral akinesia scores (*N*=26, *r*=0.5, *p*=0.009) and with the contralateral tremor scores (*N*=26, *r*=0.51, *p*=0.008). Moreover, beta power was strongly correlated with the nonlinear measure (*N*=26, *r*=0.55, *p*=0.0035). When the beta power values were included in the regression together with the nonlinear measure as explanatory variables for tremor, only the beta power effect remained significant (*p*=0.029) while the effect of the nonlinear measure was not (*p*=0.7).

### 4.3 Alpha nonlinearity in the MEG cortical sources

We also examined the nonlinear effect for the cortical sources maximally coherent with STN LFP activity. Two groups independently concluded that the synchronization between STN and cortex for PD patients occurs for alpha and beta bands (Litvak et al., 2010; Hirschmann et al., 2011).

In contrast to the STN case, for cortical signals, the nonlinear behavior was absent in the OFF drug state and appeared only ON medication. It was specific to the alpha band. The increase in nonlinear behavior was seen both in sources coherent with the STN in the beta band and those coherent in the alpha band. We present the results for signals from the alpha-coherent sources but the results for the beta-coherent sources were similar.

**Fig. 6:**
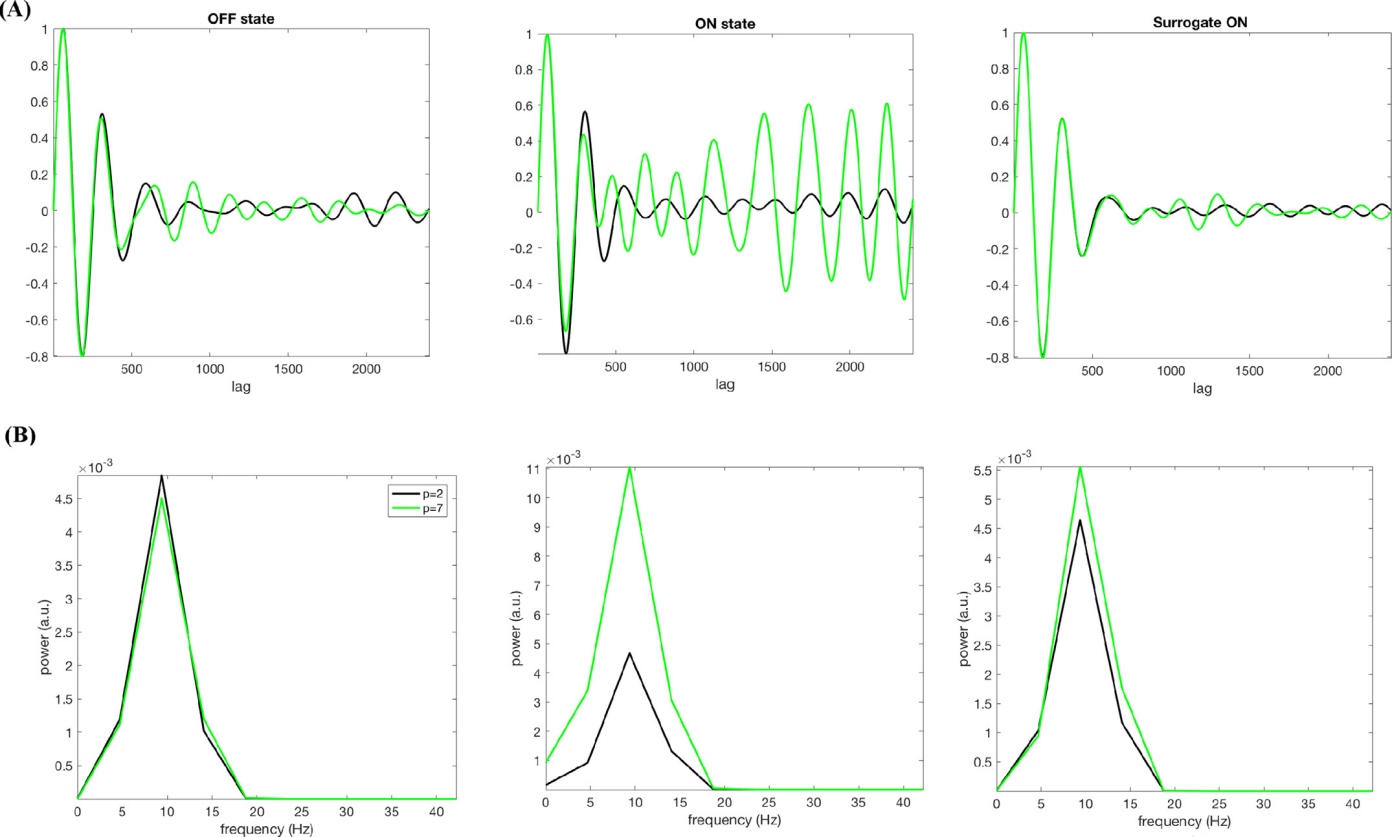
(A) nsAMDF for the degrees *p*=2 (black) and *p*=7 (green) for a cortical source OFF state, ON state and OFF state surrogate time series for a representative subject (nsAMDF bandpass filtered on the alpha band range for illustrative purposes) and (B) their corresponding spectra in the same order of states. *y*-axis is the same in all three panels.

**Fig. 7:**
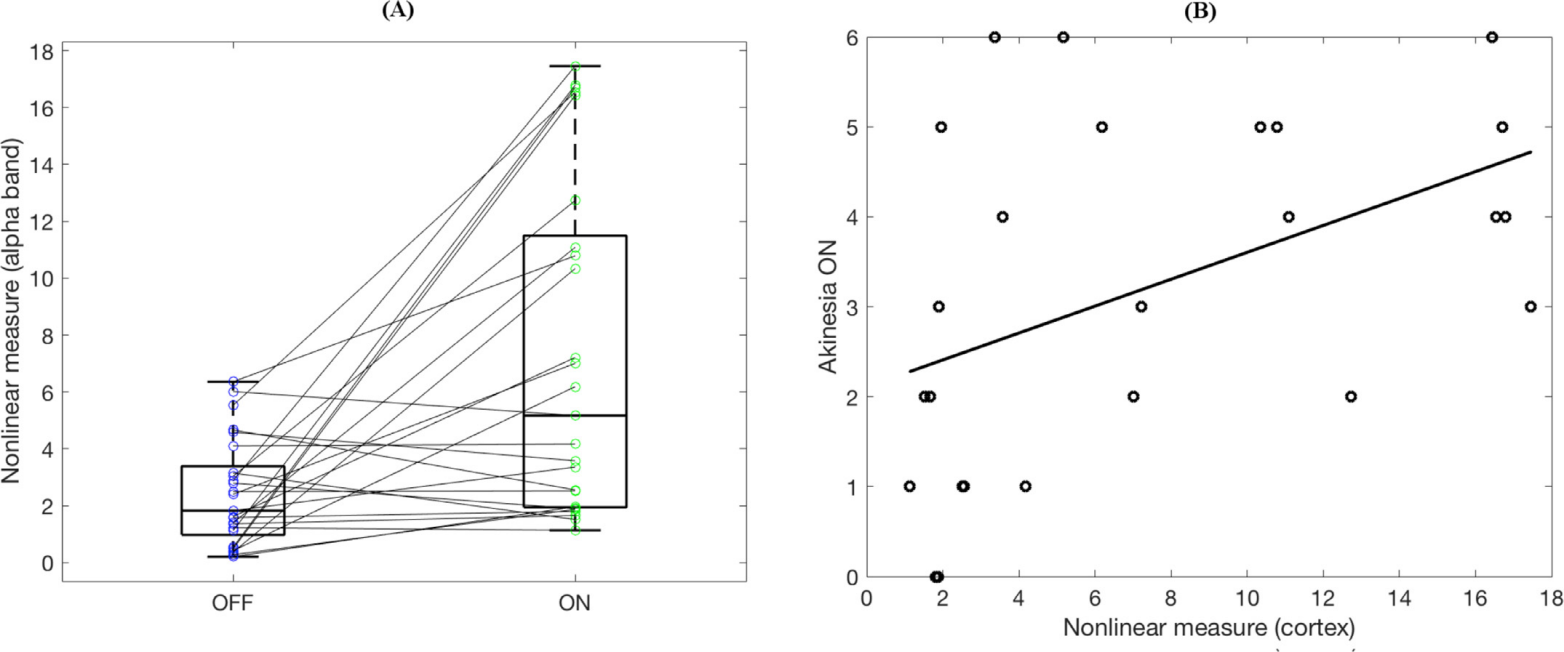
(A) The nonlinearity is higher for the medication ON state compared to OFF state (*N*=25; *p* < 6.10^−4^). (B) The nonlinear measure for the ON state correlates with the akinesia subscores (*r*=0.46, *p* = 0.02).

In order to confirm the observed cortical alpha band nonlinearity, we produced surrogate data preserving the linear second order characteristics of cortical data for the ON state. Fig. 6 depicts dopaminergically induced cortical nonlinearity for a representative subject. While the nonlinearity measure *L* is 3.03 for the OFF state (Fig. 6(A), left column), it increases up to 12.73 for the ON state (Fig. 6(A), middle column). The absence of the nonlinear effect for the OFF state and the surrogate ON data can also be observed in the frequency domain in Fig. 7(B). The alpha band nsAMDF difference for the ON state (Fig. 6, middle column) drastically diminishes for the surrogate data (Fig. 6 right column). Accordingly, the nonlinearity measure *L* for the surrogate ON state data dropped to 3.12, hence moving to a similar level to the OFF state.

Statistical analysis over the whole group (Fig. 7(A)) showed that the nonlinearity measure *L* was significantly higher for the ON state than the OFF (paired *t*-test, *N*=25; *p* < 6.10^−4^). Three nonlinear measure estimates falling out of 95 % of the range were taken as outliers and removed from the analysis. The nonlinearity effect for the ON state correlated with contralateral UPDRS akinesia scores (Fig. 7(B); Pearson's correlation, *r*=0.46, *p*=0.02). Its correlation with the tremor scores did not reach significance (*r*=0.36, *p*=0.08).

To compare the clinical correlations of the nonlinear measure with alpha power, we also computed alpha power estimates by averaging the corrected log-spectra (see Section 2) for the ON medication state. The resulting values were not significantly correlated with contralateral akinesia scores (*N*=25, r=0.36, *p*=0.07) nor with contralateral tremor scores (*N*=25, *r*=0.04, *p*=0.84). Alpha power was also not correlated with the nonlinear measure (*N*=25, *r*=0.01, *p*=0.95). When the alpha power values were included in the regression together with the nonlinear measure as explanatory variables for akinesia, the effect of the nonlinear measure was significant (*p*=0.016), while the alpha power effect did not reach significance (*p*=0.055). The r-squared for the combined model was 0.34 compared to 0.13 for power alone and 0.21 for the nonlinear measure alone.

## 5 Discussion

Linear properties of brain signals are simple to extract and interpret. However, as the brain is notoriously complex and nonlinear, the description of brain mechanisms will be incomplete without including nonlinear and nonstationary properties. A vast majority of nonlinear methods suffer from computational complexity. This makes the practitioners shy away from applying these methods. When increasing the order by one in higher order statistics (e.g., bispectrum) the order of complexity is multiplied by the data length (Nikias and Mendel, 1993). Other promising methods such as Lainscek et al. (2013a) are iterative and computationally expensive.

Here, we proposed a new method whose main advantages are simplicity in implementation and efficiency when applying to real biomedical data. It simply takes into account the difference between degrees of nsAMDF, which reveals the nonlinear autocorrelative memory inherent within the signal. Moreover, its autocorrelative character makes the analysis interpretability rather straightforward, which is one of the desirable properties for nonlinear methods in real data applications. Another desired feature of our method is that it is essentially temporal, i.e., it requires neither *a priori* selection of a frequency band, nor a subsequent band-pass filtering. After the examination of *L*, Fourier analysis can be used to find the bands involved in nonlinear phenomena.

When we applied the proposed method to PD LFP-MEG data, our analysis revealed, to our knowledge for the first time in PD literature, the inherent nonlinearity in the PD data for subcortical high beta (20–30 Hz) and cortical alpha (8–12 Hz) activity. In the STN, high beta nonlinearity was prominent in the OFF medication state and was significantly correlated with low beta oscillatory power. It might, therefore, be related to some property of the beta oscillation such as the waveform shape or burst statistic (Tinkhauser et al., 2017). However, our analyses have failed to reveal such a relationship to date. We would like to note that, after high-pass filtering STN LFP data above 10 Hz, it did not exhibit any nonlinear effects at the high beta band for the OFF state. This suggests that the nonlinear component could be a result of interactions of oscillations at various frequencies lower than 10 Hz, possibly including the tremor frequency (Hirschmann et al., 2013b), which might explain why the nonlinear measure was correlated with tremor, but not with akinesia.

We also investigated nonlinear features in the cortical sources reconstructed with beamforming inverse technique applied on the MEG data of the patients. Since the nonlinearity metric implied changes in the alpha band for the individual cases, we selected the source time series that are maximally coherent with STN LFP data for this band. However, there was no qualitative difference in results, when the sources maximally coherent with the STN in the beta band were used (data not shown). This might be because nonlinear effects in the alpha band are observed in multiple cortical areas or because the beamformer cannot perfectly separate the activities of different cortical sources. To better understand the origins of the nonlinear effect it will be necessary to compute it for the whole brain volume and look at the topography, but this was outside the scope of this first proof-of-principle study. Also clearer results might be obtained with PD patients who do not have DBS leads in their head and can be better positioned in the MEG scanner.

Contrary to the case for STN LFP, the nonlinearity within cortical sources was found in the ON medication state and was constrained to the alpha band. Moreover, it correlated with contralateral hemibody akinesia scores and this correlation could not be explained by alpha power. Previous studies showed that dopaminergic medication has an effect on cortical oscillations (Melgari et al., 2014) and there is evidence that at least in advanced PD patients such as our cohort, the medication acts to increase beta power, which is the opposite of its effect in the basal ganglia (George et al., 2013; Heinrichs-Graham et al. 2014). Melgari et al. (2014) showed an increase in both alpha and beta EEG power induced by dopamine and a correlation between the beta increase and clinical improvement. Whether these power changes are related to the nonlinear effect we found requires a more careful study for which our cohort of DBS patients is not ideally suited.

Another possibly related phenomenon was shown by Oswal et al. (2013) whose patient cohort largely overlapped with ours. In that study alpha coherence between the STN and cortical sources (defined the same way as in our study) was decreased during movement. This effect was enhanced by dopamine and the degree of enhancement correlated with improvement in akinesia and rigidity. Timmermann et al. (2004) showed that stimulation at the alpha central frequency of 10 Hz increases akinetic severity. Thus, there is evidence that cortical processes involving specifically the alpha band could be related to impairment in PD and perhaps the effect we found is even related to the cortico-subtalamic loops but to really gain insight into that, our method should be extended to pairs of time-series.

The computational efficiency of our method made it possible to easily apply it at the group level and reveal potentially promising clinical correlations. The effects appear to be related to classically defined frequency bands previously shown to be involved in PD pathology. However, the exact relation between the nonlinear (*p*=7) and conventional (*p*=2) spectra requires more careful study and modeling of the processes generating these effects.

Closed-loop adaptive DBS devices are currently an active field of study (Jensen et al., 2008; Rossi et al., 2007; Brittain et al., 2010; Beudel and Brown, 2016) and are expected to enter into clinical use in the near future. They infer the clinical state from online analysis of LFP signals from the stimulation target and possibly other measures (e.g., accelerometry) and change the DBS parameters adaptively to maximize stimulation benefit, increase battery life and reduce side effects. Since our measure can be efficiently estimated over short time from single channel data, it could well be a promising candidate for use in these closed-loop devices. Also our measure is sensitive to the cortical effects of dopamine and can be estimated non-invasively perhaps even with single-channel EEG. It can, therefore, possibly be used for optimization of drug treatment. Hence, the proposed method not only holds promise for understanding PD mechanisms but also offers a potential tool of practical clinical utility. Although in the current study, the suggested metric was applied to signals from PD patients, we would like to underline that it is straightforward to apply to any single time series.

## Author credits

**Tolga Esat Özkurt:** Conceptualization, Methodology, Formal Analysis, Software, Writing - Original Draft. **Harith Akram:** Resources, Writing - review & editing. **Ludvic Zrinzo**: Resources, Writing - review & editing. **Patricia Limousin**: Validation, Resources, Writing - review & editing. **Tom Foltynie**: Resources, Writing - review & editing. **Ashwini Oswal**: Data Curation, Investigation, Writing - review & editing. **Vladimir Litvak**: Investigation, Data Curation, Validation, Software, Visualization, Writing - Review & Editing.

## Appendix Supplementary materials

Image, image 1
