# Supplementary figures and images for: Identification of nonlinear features in cortical and subcortical signals of Parkinson's Disease patients via a novel efficient measure
Source: Neuroimage. 2020 Dec;223:117356. doi: 10.1016/j.neuroimage.2020.117356 (PMC8417768; doi:10.1016/j.neuroimage.2020.117356)

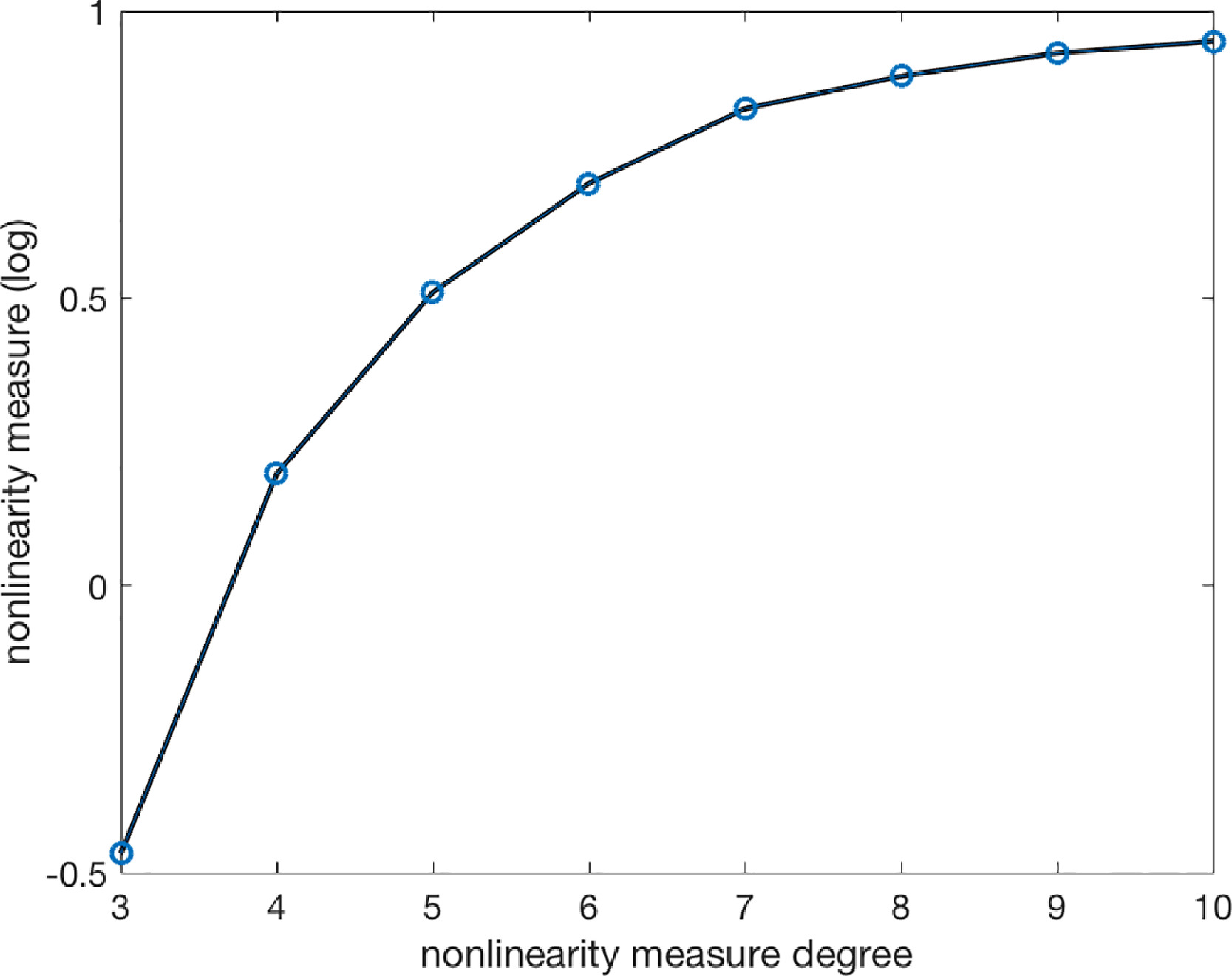

Supplement: Supplementary file 1 [file mmc1.jpg]
